# Molecular study of KIT gene that is related to drug resistance in chronic myeloid leukemia patients

**DOI:** 10.1016/j.jgeb.2026.100769

**Published:** 2026-07-20

**Authors:** Asrar K. Salman, Ahmed E. Dhamad

**Affiliations:** a Department of Biology, College of Sciences, Wasit University, Iraq; b Department of Pathological Analyses, College of Applied Medical Sciences, University of Kerbala, Iraq

**Keywords:** Chronic myeloid leukemia (CML), KIT gene, Mutations, Tyrosine kinase inhibitors (TKIs)

## Abstract

The KIT gene, known as proto-oncogene c-Kit, encodes a receptor tyrosine kinase and plays a crucial role in cellular processes through activation that initiates signal transduction pathways. The KIT protein includes five extracellular domains, a kinase domain, a transmembrane helix, and a juxta-membrane domain. The study investigated these domains in chronic myeloid leukemia (CML) patients, revealing insights that enhance clinical protocols for tyrosine kinase inhibitors used in targeted therapy for hematological malignancies. However, mutations in the kinase domain present challenges by impairing drug binding. In this exploratory investigation, mainly through PCR and Sanger sequencing techniques, 50 samples (30 patients and 20 controls) were utilized. A total of 27 mutations were identified. While the following mutations (G753C, G538C, A415T, and A404G) were the most frequent, (G814C, G858T, G876T, and G880A) were only found in control cohort. Interestingly, many unique mutations were detected in the Iraqi population, suggesting specific genetic polymorphisms. Furthermore, the data showed that younger patients had better responses to imatinib with fewer dangerous mutations, while middle-aged patients faced significant resistance, which correlated with weight gain and obesity. Elderly individuals displayed a higher mutation burden, necessitating multiple treatment changes. Despite limitations, this exploratory study stressed the importance of considering patients' genetic backgrounds and BMIs to improve therapeutic outcomes for CML patients.

## 1 Introduction

**Fig. 1:**
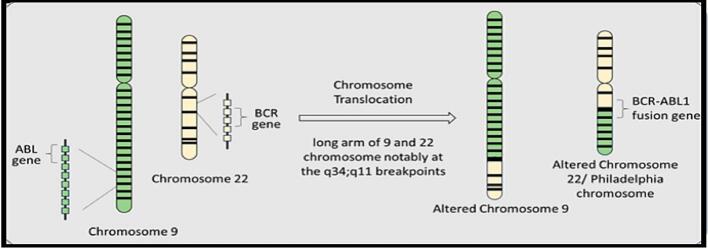
Philadelphia chromosome: Occurrence of the Philadelphia chromosome results from a chromosomal translocation event where the genetic material is exchanged between chromosome 9 and chromosome 22. Adapted from Kausar, Anwar, & Khan, 2025.^8^

**Fig. 2:**
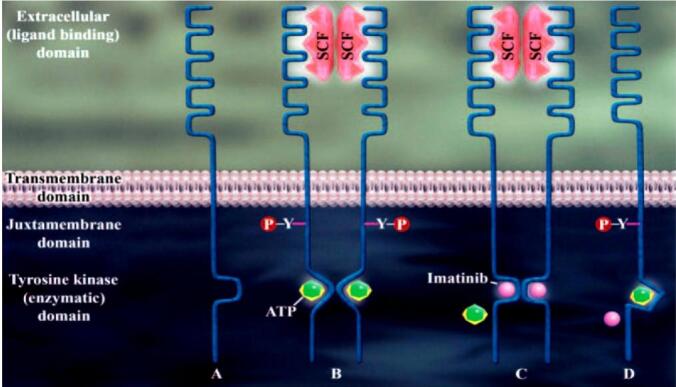
The Kit monomer implements an inactive configuration in the absence of its ligand (A). The cross-linking of Kit by SCF generates a structural alteration in the kinase domain, facilitating ATP binding and subsequent tyrosine phosphorylation (B). Imatinib attaches to the inactive conformation of Kit and prevents the binding of adenosine triphosphate (ATP). Mutations in the enzymatic site or tyrosine kinase domain of the protein provide resistance to imatinib by obstructing its association to Kit (D). Adapted from Arranz, Salazar, & Bote (2022).

Chronic Myeloid Leukemia (CML) is a clonal myeloproliferative disorder driven by the t(9;22) (q34;q11) translocation, which creates the Philadelphia chromosome and the subsequent BCR:ABL1 fusion protein (Fig. 1).^1^ This chimeric protein functions as a constitutively active tyrosine kinase that drives leukemogenesis by promoting cell survival and suppressing apoptosis through continuous auto-phosphorylation.^1^, ^2^ While Tyrosine Kinase Inhibitors (TKIs) like Imatinib have revolutionized treatment by binding to the ATP-binding domain and locking the enzyme in an inactive conformation,^3^ clinical challenges such as Imatinib resistance and failure to achieve Complete Hematological Response (CHR) persist.^2^ These challenges are often compounded by secondary mutations, such as those in the c-Kit receptor. Specifically, mutations in Exon 11 abolish the protein's autoinhibitory constraints, while Exon 9 mutations increase dimerization affinity, both leading to ligand-independent signaling that further dysregulates hematopoiesis and cell proliferation.^4^, ^5^ Despite a major clinical advance in the treatment of CML, Imatinib resistance has become a challenging problem. The existence of patients resistant to Imatinib was evident soon after the introduction of the drug into clinical practice.^6^ Initial responses were lower in patients with advanced-phase disease (Fig. 2). Studies with second-generation TKIs like Nilotinib and dasatinib have also shown superior efficacy to Imatinib in newly diagnosed CML, inducing faster and higher rates of molecular response. Therefore, both drugs are approved by the Food and Drug Administration for use in patients with newly diagnosed CML in chronic phase (CML-CP).^7^

In this preliminary study, the aims were to evaluate the rarity of KIT gene mutations and the genetic resistance to drugs (e.g., imatinib and dasatinib) landscape in Iraqi CML patients and to enhance understanding of how factors such as age, blood types, BMI, and TKI response contribute to the molecular complexity of the disease in this specific group.

## 2 Materials and methods

### 2.1 Sample collection

In the first half of 2025, 50 peripheral blood samples were taken as part of standard complete blood count (CBC) assessments. Thirty patients with CML made up the study cohort. They were then divided into two equal subgroups: those on medication (W.D., *n* = 15) and those not on medication (W, n = 15). For comparative study, a control group of 20 healthy people was added. Scorpio Labs and Cellavision, two sophisticated digital morphology platforms, were used for morphological evaluations. To assess hematological differences among the study groups, all specimens were obtained from the laboratory of the Wasit Specialized Oncology Centre in the Wasit Governorate.

### 2.2 DNA extraction

In accordance with the manufacturer's instructions, DNA was extracted from the blood of CML patients as shown in the subsequent steps. To put it briefly, the samples were incubated at 60 °C after 200 μl of GST buffer and 20 μl of proteinase K were added. The supernatant was then transferred to a brand-new 1.5 ml microcentrifuge tube, combined with 200 μl of GSB buffer, and added to the GS column. Following centrifugation, 400 μl of Buffer W1 was used to wash the samples into the GS column. Lastly, 70 μl of hot elution buffer was added to the column's core to elute the DNA, which was then centrifuged for three minutes at room temperature at 14000 xg. The eluted DNA was stored at −20 °C until being used.

### 2.3 KIT-gene amplification and confirmation

The extracted DNA of the CML patient was utilized as a template for PCR amplification. To amplify the JM region (1112 bp) (NCBI Reference Sequence: NC_000004.12), a standard PCR with specific primers was run. The reaction mixture contained 25 μL of Taq polymerase master mix (Promega cat#M7822), 19 μL nuclease-free water, 2 μL forward primer (DLP·F: 5′- CAGTTTGGGACTGAGTGGCT-3′),2 μL reverse primer (DLP.R: 5′- GCAAGAGAGAACAACAGTCTGG-3′) and 2 μL of DNA. Amplification was carried out using a thermal cycler (Multigene Optima Thermal Cycler) with the following protocol. The initial denaturation was at 95 °C for 3 min, followed by 40 cycles consisting of denaturation at 95 °C for 30 s, primer annealing at 57 °C for 30 s, and extension at 72 °C for 1 min. The final step was at 72 °C for 5 min. Amplicons were saved at −20 °C until they were still used.

### 2.4 Sanger sequencing

A Gel/PCR DNA Fragments Extraction KIT (Gene Aid/Cat#DF100) was used to purify the most prevalent among the mutations observed, suggesting that alterations in the juxta membrane JM region may band. The purified products were then sent to sequencing facilities at Microgen Corporation in Korea. The Sanger sequencing approach was applied. The sequencing data were subsequently analyzed using Bloedite software ver.7.7.1 to reveal that the mutations were the most prevalent among the mutations observed, suggesting alterations in the kit gene.

## 3 Results and discussion

### 3.1 Hematological and therapeutical characteristics

Hematological techniques were used to assess variables of interest as possible predictors, which is highlighted by the distribution of disease phases, risk classification, therapy types, and patient outcomes. Twenty healthy control samples include 10 males and 10 females, with ages from 22 to 66 and weights between 70 and 95. Most females exhibit higher CBC values, with an average of 7.88, compared to 6.73 in males. While average age and weight are very similar between the sexes The ages in the table range from 20 years (the smallest sample C19) to 66 years (the largest sample C9), with a high degree of convergence in the overall average age around 40. Blood type B+ is the most frequent in the samples (Table 1).

**Table 1:** Questionnaire data of controls.

		NO	Sample code	Age	Gender	BMI⁎ (Kg/m^**2**^)	(CBC) 10^3^/ μL	(WBC) 10^3^/ μL	Blood group
					RBC	Hb	PLT		
1	C1	37	Male	29.4	4.58	13.0	174	8.4	B+
2	C2	22	Male	22.5	5.28	15.5	168	6.3	B+
3	C3	50	Female	29.4	3.89	11.2	266	5.3	B+
4	C4	40	Female	24.9	4.32	11.9	219	7.7	B+
5	C5	40	Female	31.1	4.78	12.8	221	6.1	B+
6	C6	43	Female	24.2	4,57	11.8	322	8.78	O+
7	C7	43	Male	32.9	5.10	15.2	162	6.3	B+
8	C8	50	Female	31.1	4.55	11.3	257	6.1	B+
9	C9	66	Male	31.1	4.90	14.0	175	6.3	B+
10	C10	42	Female	27	4.69	12.5	208	9.5	AB+
11	C11	25	Female	20.1	4.30	11.7	328	9.2	B+
12	C12	52	Female	26.3	5.11	11.9	364	8.2	O+
13	C13	30	Female	23.5	4.67	12.4	329	8.1	O+
14	C14	41	Male	26.3	5.07	15.3	222	7.3	B+
15	C15	55	Male	26.3	4.59	12.2	262	7.3	O+
16	C16	42	Male	26.3	4.90	15.6	164	5.48	O+
17	C17	32	Female	26	4.11	11.7	264	9.8	B+
18	C18	40	Male	27	5.21	15.2	165	6.8	B+
19	C19	20	Male	22.5	5.37	13.3	279	6.4	O+
20	C20	42	Male	26.3	/	/	/	7.6	O+

⁎ The Body Mass Index (BMI) Normal (18.5–25), Overweight (25–30), Obesity (30–35).

Increasing the inhibition of TKI drugs (Nilotinib, Bosutinib) reflects the need for more complex drug management in these categories; therefore, the improvement in survival among older patients is lower than that in younger patients (20–39 years), a trend observed across all life phases. The average age of patients tends towards middle age (47 years). There were no significant differences in weight change between the categories (W, WD). We note that the average number of white blood cells is similar between the sexes, with a value of (57.62) for females and (19.58) for males. This indicates that the WBC level does not differ significantly between the sexes, but rather is affected by the clinical condition (phase) related to splenomegaly. Patients in the (chronic phase) have lower WBC levels, indicating that this category represents cases well controlled therapeutically; 13 cases (7 females and 6 males) were treated with imatinib. Increase in WBC in a patient who suffers from splenomegaly (7 cases) in the accelerated phase, with 7(4 females,3 males). These observed incidences might be one of the causes to the disease progression. As gender shows females have more variety for drug use and a shift from Imatinib to TKs' second generation (Nilotinib, Bosutinib) (17).

(56.7%), compared with men (13) (43.3%), which leads to treatment response differences being seen. The frequency of leukemia is concentrated in the Al-Hay area, with 8 cases compared to 14 different addresses, and blood group O+ is more frequent among patients (11 cases). About a quarter of TKI-treated patients develop TKI resistance within 5 years of treatment duration. Therefore, appears to be a mutation-resistant drug (Table 1, Table 2).

**Table 2:** Questionnaire data of CML patients.⁎

No	Sample Code	Age	Gender	BMI (Kg/m^2^)	CBC test RBC/Hb/PLT	WBC)10^3/^μL)	Blood group	Type of drug	Duration of drug
1	W1	30	Female	22.7	3.61/11.9/130	19.0–14.2	O+	Imatinib	2022–2025
2	W2	42	Female	21.6	3.19/8.1/99	19. 5–15.6	O+	Nilotinib	2023–2025
3	W4	46	Female	29.3	3.34/10.5/153	18.3–14.6	A+	Imatinib - Nilotinib	2020–2025
4	W5	19	Female	22.7	4.78/12.1/233	8. 6–6. 9	O+	Imatinib	2024–2025
5	W6	61	Female	35.2	3.83/12.0/183	7.9–8.8	O+	Imatinib	2020–2025
6	W7	30	Female	23	4.75/10.3/226	8.1–6.6	O+	Imatinib	2024–2025
7	W8	35	Female	19.4	3.25/10.4/169	12.4–13.7	B+	Imatinib - Nilotinib	2023–2025
8	W9	73	Male	34.8	3.78/10.7/432	6.0–7.6	AB+	Imatinib	2023–2025
9	W10	54	Female	29.7	3.90/11.5/244	4.9–9.0	AB+	Imatinib	2023–2025
10	W11	55	Female	34	4.71/14.0/220	8.0–12.4	A+	Imatinib - Nilotinib	2024–2025
11	W12	43	Female	24.6	6.06/12.4/322	4.3–3.8	B+	Imatinib	2023–2025
12	W13	30	Male	21.9	5.59/11.0/237	8.0–5.2	AB+	Imatinib	2023–2025
13	W14	51	Male	23,8	5.35/11.1/411	69.5–21.4	AB-	Imatinib - Nilotinib	2022–2025
14	W15	22	Male	22.2	6.52/12.2/303	8.14–7.3	O+	Imatinib	2020–2025
15	W16	65	Male	34.4	4.55/14.5/150	7.8–16.9	O+	Imatinib - Nilotinib	2017–2025
16	WD1	65	Male	34.4	2.72/8.9/35	7.8–6.9	O+	Imatinib	2017–2025
17	WD4	45	Male	30.1	5.40/14.8/136	9.6–7.8	O+	Nilotinib	2019–2025
18	WD5	56	Female	31.3	3.97/11.1/169	9.9–5.5	B-	Imatinib	2018–2025
19	WD6	59	Male	27.3	5.42/14.9/308	57.5–28.9	AB+	Imatinib	2020–2025
20	WD7	59	Male	34.4	4.64/14.3/237	15.3–6.10	AB+	Imatinib	2015–2025
21	WD8	39	Male	29.3	3055/10.6/55	19.8–15.4	B+	Imatinib	2020–2025
22	WD9	78	Male	27.8	4.84/13.2/174	16.6–15.7	O+	Imatinib	2024–2025
23	WD10	36	Male	23.1	4.26/12.4/1060	11–17.2	A+	Imatinib-bosutinib	2018–2025
24	WD11	77	Male	23.8	4.09/12.6/162	18.4–15.7	B+	Imatinib - bosutinib	2013–2025
25	WD12	49	Female	22.5	3.82/11.6/129	5.9–5.1	O+	Imatinib	2015–2025
26	WD13	25	Female	17	3.20/9.3/130	10.5–19.5	AB+	Imatinib- bosutinib	2010–2025
27	WD14	50	Male	26.9	3.81/13.5/247	8.4–18.5	A+	Imatinib – Nilotinib - Bosutinib	2013–2025
28	WD15	45	Female	22.2	1.95/5.4/24	16.7–17.8	A+	Imatinib - Nilotinib	2015–2025
29	WD16	50	Female	23.8	4.01/12.9/271	8.7–12.9	O+	Imatinib	2008–2025
30	WD17	53	Female	18.5	3.56/10.5/163	14.1–16.1	A+	Bosutinib - Imatinib	2017–2025

⁎ The Body Mass Index (BMI) Normal (18.5–25), Overweight (25–30), Obesity (30–35) (Calculator.net, n.d.) ^[23]^.

### 3.2 Mutational profile of KIT gene and TKIs

**Fig. 3:**
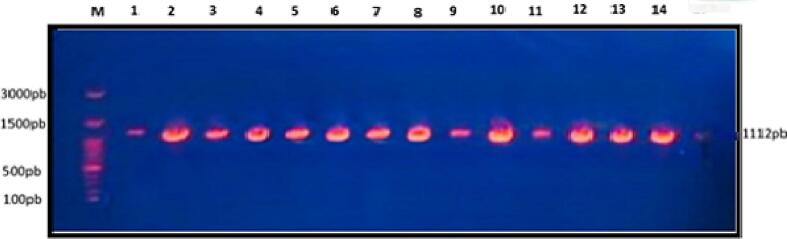
Agarose gel electrophoresis: 5 μL of the kit PCR product was mixed with 1 μL of loading dye and loaded into the gel wells. M: A 100 bp DNA marker. Lanes 1 to 14 were kit-positive.

Adequate amounts of and cleaned DNA were gained from all samples (30 CML and 20 Control group) after using the gSYNCTM DNA extraction kit. To amplify the region, a standard PCR with specific primers was run. The PCR products were resolved by a 0.5% agarose gel, and bands (juxta membrane JM region, amplicon 1112 bp) were shown in Fig. 3. Although there are many obstacles to extracting DNA from CML samples and amplifying genes, our results showed a 97% success rate. The following reasons could account for the 3% unsuccessful rate. The effects of Elution and crosslinking buffers on the quality of genomic materials. Technical issues, including insufficient Elution buffer and storage at −20 °C could alter the DNA quality.^9^, ^10^ To investigate mutations in the juxta membrane domain (JMD) region, the amplicons were cleaned with the Gel/PCR DNA Fragments Extraction kit and sent to Sanger sequencing facilities (Microgen, Korea). The obtained sequence results were analyzed by BioEdit software.

CML stands as a distinct cancer type, especially when considering its molecular genetic underpinnings. It is a malignant hematologic disorder characterized by a proliferation of abnormal white cells that infiltrate the bone marrow, peripheral blood and organs. Central to this disease is a specific mutation.^11^ A consistent cytogenetic abnormality characterizes CML, the Philadelphia chromosome (pH), which results in a unique gene product, the BCR-ABL1 fusion. The deregulated tyrosine kinase activity is the hallmark of CML, as it is responsible for myeloid cell proliferation, causing Mutations in the DNA.^12^ These mutations may occur spontaneously or as a result of exposure to radiation or carcinogenic substances and are influenced by genetic factors such as family history, blood disorder and genetic disorder.^9^

Table 3 showed changes of nucleotide bases replacement in G/Tin samples (W1, W2, W5, W8, W11, W12, W13, W14, W15), identified mutations G/T, were within the phosphorylation site of JM regions of the KIT gene of the patient without(W), the.

**Table 3:** Identified mutations in the KIT gene in both CML patients and controls.

No.	Sample	Mutation	Chang Nucleotide	Gene Region	Position in the whole KIT	Position In JM	Type of Mutations
1	WD1, WD2, WD7, WD8, WD9, WD13, WD20	T350G	G/T	CATGA	69,541	350	Substitution
2	W4, W5, W8, W11, W14	G351T	G/T	TATCC	69,541	351	Substitution
3	C2, C3, C12, C13, C18, C20	C403A⁎	C/A	CCAGG	69,593	403	Substitution
	W1, W4, W6, W8, W11, W15						
	WD1, WD2, WD8, WD13, WD20`						
	WD3, WD7, WD10, WD12, WD13		C/G				
4	C2, C3, C11, C12, C13, C18, C20	G404A⁎	G/A	CAGGA	69,594	404	Substitution
	W1, W4, W6, W8, W11, W16						
	WD1, WD5, WD8, WD10, WD12, WD13, WD18, WD20						
	WD2, WD3, WD7, WD11, WD13, WD19		C/A	CACGG			
5	W1, W2, W10, W13	G405A	G/A	CAAGG	69,595	405	Substitution
			G/C	CACGG			
6	C3, C5, C10, C12, C13, C15, C16, C20,	A415T⁎	A/T	CATGG	69,605	415	Substitution
	W1, W5, W10, W12, W13, W16						
	WD1, WD2, WD3, WD5, WD6, WD7, WD8, WD12						
7	C1, C2, C4, C13, C15, C16, C17, C18, C20	G420A⁎	G/A	TCGCC	69,610	420	Substitution
	W1, W5, W10, W12						
	WD1, WD6, WD9, WD11, WD18						
8	C1	DL486T	DL/T	CCTGA	69,676	486	Deletion
9	W4	C503T	C/T	GGCCT	69,689	503	Substitution
10	W7	A503T	A/T	TTTAT	69,689	503	Substitution
11	C4, C6, C11, C12, C14, C15, C16, C20	T505G	T/G	GTGAG	69,695	505	Substitution
	W2, W5, W10, W12, W16						
	WD2, WD5, WD6, WD9, WD11, WD19						
12	C18	DL516C	DL/C	TGCGC	69,706	516	Deletion
13	C1	DL521T	DL/T	TTTGA	69,711	521	Deletion
14	C1	DL529T	DL/T	GGTTT	69,719	529	Deletion
15	C1, C2, C3, C4, C5, C6, C7, C8, C9, C10, C12, C13, C14, C16, C17, C18, C19	G538C	G/C	TACAG	69,728	538	Substitution
	W2, W5, W10, W12, W13						
	WD2, WD6, WD8						
16	C1, C3, C4, C5, C6, C8, C9, C11, C13,C14, C15, C16, C17, C18, C19, C20	G547T	G/T	TCTTA	69,737	547	Substitution
	W2, W5, W10, W12, W13, W16						
	WD1, WD6, WD8, WD19						
17	C1, C2, C3, C4, C6, C7, C8, C9, C10, C13, C14, C15, C16, C17, C18, C19	G550C	G/C	AGCGA	69,740	550	Substitution
	W5, W10, W12						
18	C15, C20	A579T	A/T	TTTAT	69,769	579	Substitution
	W2, W5, W8, W10, W13, W14						
	WD5, WD9, WD11, WD13, WD19						
19	C15, C20	A580C	A/C	TTCAT	69,770	580	Substitution
	W2, W14, W15						
	WD2, WD7, WD9, WD13						
20	W2, W6, W8, W11, W14, W15, W16	T587C⁎	T/C	TTCTT	69,777	587	Substitution
21	C1, C2, C8, C12, C14, C16, C19, C20	A628G⁎	A/G	CCGCC	69,818	628	Substitution
	W2, W12, W13						
	WD1, WD5, WD6, WD13						
22	C1, C2, C3, C4, C5, C6, C7, C8, C9, C10, C12, C13, C14, C16, C17, C18, C19, C20	G735C⁎	G/C	AACTC	69,925	735	Substitution
	W5, W8, W10, W12, W13, W14, W15						
	WD1, WD5, WD6, WD19, WD20						
23	C1,C2,C3,C4,C5,C6,C7,C8,C9,C10,C12,C13,C14,C16,C18,C19	G813C⁎	G/C	TCCGT	70,003	813	Substitution
24	W1,W2,W6,W8,W10,W11,W14,W16	T816G	T/G	TTGCC	70,006	816	Substitution
25	C2,C3,C4,C7,C8,C9,C10,C13,C14,C17,C18,C19,C20	G858T⁎	G/T	TATCG	70,048	858	Substitution
26	C2,C3,C4,C6,C8,C9,C10,C13,C17,C18,C19	G876T⁎	G/T	ACTGA	70,066	876	Substitution
27	C1,C2,C3,C4,C5,C6,C7,C8,C9,C10,C11,C12,C13,C14,C15,C16,C17,C18,C19,C20	G880A⁎	G/A	ACAGG	70,070	880	Substitution

⁎ Unique mutation.

drug Imatinib – Nilotinib during (2-3 year) of treatment.

Nearly all of the samples in this category (C1–C20) exhibit the phenomenon of mutational matching, but deletion, exclusively in group C (samples C1 and C18) at positions 486, 516, 521, and 529 (Table 3, Table 4). This deletion is considered one of the most serious types of mutation, as it can lead to a frameshift, altering the entire KIT protein. The most prevalent variants are located at locations 547 (T547G), 880 (A880G), and 735 (C735G). Nearly every sample in the cohort has these mutations, indicating a consistent genotype or exposure to a common mutagenic agent. A distinct mutation transversion between the purine-pyrimidine (A-G), (C-G), and (T-G) was discovered. It is seen as more hazardous since transversion, a region rich in C and G, directly affects protein synthesis in addition to changing the gene's function (C1–C20). There is a large concentration of substitutions at positions 735, 880, 505, and 547 in C as well as in W and WD, indicating that it is either a widespread SNP single-nucleotide polymorphism or a silent mutation (Table 5). All study categories (Control C, without drug W, with drug WD) share the mutation G404A. A403C is an unstable mutation in all groups, but WD displayed a G/C variant. In C and W, the mutation is common as G/A, while WD is unusual in possessing C/A. This is explained by the use of imatinib and nilotinib to treat CML in this population.

**Table 4:** Distribution of mutations according to age.

Group	Age	Sample code
Group 1	Youth (18–35 years)	W5,W15,WD13,W1,W7,W13,W8
Group 2	Mid-Life (36–55 Years)	WD10,WD8,W2,W12,WD4,WD15,W4,WD12,WD14,WD16,W14,WD17,W10,W11
Group 3	Seniors (56–78 Years)	WD5,WD6,WD7,W6,WD1,W9,WD11,WD9

**Table 5:** Frequency of mutations in the KIT gene.

No.	Mutations	Location	Frequency	Percentage %
1	G880A	CD	20	40%
2	G735C	CD	30	60%
4	G538C	PH	25	50%
5	T816G	CD	8	16%
6	A415T	PH	22	44%
7	G351T	KD	5	10%
8	A404G	PH	21	42%
9	A404C	PH	6	12%
10	G350T	PH	7	14%
11	A579T	PH	13	26%
12	A505G	PH	19	38%
13	DL486T	PH	1	2%
14	DL521T	PH	1	2%
15	DL529T	PH	1	2%
16	DL516C	PH	1	2%
17	C403A	PH	17	34%
18	C403G	PH	5	10%
19	G405G	PH	2	4%
20	A420G	PH	18	36%
21	T503C	PH	1	2%
22	A503T	PH	1	2%
23	G550C	PH	19	38%
24	A580C	PH	9	18%
25	T587C	PH	7	14%
26	G628A	PH	18	36%
27	G813C	PH	16	32%
28	G858T	PH	13	26%
29	G876T	PH	11	22%

Catalytic domain (CD); kinase domain (KD); Phosphate domain (PH).

Point mutations frequently have the C735G mutation from the kinase domain (KD) in the Bcr or Abl areas have been found, suggesting possible imatinib resistance. This is consistent with discoveries of KD mutations in a significant number of imatinib-naïve patients with advanced-phase CML, and even in the control group. Approximately 98% of all groups, including G/A, T/G, and C/A, have substitution mutations, which are the most prevalent. This suggests that single amino acid mutations, sometimes known as point mutations, are the primary cause of genetic alterations. These changes may have an impact on chromosome translocation due to their high frequency, particularly at positions 547, 550, and 580. C-G pair-rich regions, such as T/G, can disrupt DNA's stability, resulting in unstable mutations and challenges with DNA repair. The T351G mutation is prevalent in imatinib-resistant CML patients and significantly affects treatment responses. a patient treated with nilotinib, particularly those who had the mutation. The mutation often emerged during treatment, highlighting its role in resistance. Additionally, some patients exhibited a response to nilotinib during relapse but harbored another mutation, G 816 T. Mutations at codon 351 were also linked to either recurrence or treatment resistance. These findings emphasize the need for alternative therapies to address resistance in Ph + leukemia, particularly concerning the T315I mutation's impact on drug binding. These transversion mutations (T/G, C-G) can result in defective protein alterations, including at sensitive phosphorylation sites (e.g., BCR-ABL). Furthermore, insertions and deletions may take place, which would hinder protein inhibition and drug binding.^13^ Consequently, the illness persists as leukemia cells divide and become resistant to drugs.^14^ Control(C) group: a mutation such as A880G covers 100% of the C samples (C1 to C20), and represents only 40% of the total trial 50. This demonstrates that this mutation is a “genetic fingerprint” that distinguishes group C from the other groups (W and WD) The Without drug (W) group is defined by the G816T mutation, present in 16% of samples and serving as a strong genetic indicator for this group. Mutations at this site (including the well-known D816V mutation) were observed frequently in increasingly aggressive resistance to the conventional treatment, which may lead clinicians to consider third-generation therapies or bone marrow transplantation.

This mutation corresponds to the KIT Asp816 mutation found in humans. In cases of core-binding factor CML, an increased copy number of the mutated Asp816Tyr/Val c-KIT allele results from non-random duplication of chromosome 4, which harbors the c-KIT gene.^15^ The methionine-to-threonine mutation G351T at codon 351 [M351T] was observed in these five samples, and treatment was changed from imatinib to nilotinib (W4, W5, W8, W11, W14). ABL-kinase-domain mutations, also known as “driver mutations,” mediate resistance to imatinib and encourage the development of a second generation of TKIs capable of inhibiting these mutant proteins. Multiple factors may contribute to imatinib resistance, including altered intracellular drug availability caused by drug influx and efflux transporters.^16^ Overexpression of the adenosine triphosphate (ATP)-binding cassette (ABC) subfamily B, member 1 (ABCB1) gene, which encodes the P-glycoprotein drug efflux pump, has been observed in imatinib. The most common mutation in exon 9 is the duplication of the amino acid.^17^ Additional rare mutations have been described in patients, including an N505I, Yr503 substitution and a duplication of amino acids Y503 (Dup Y503), which reshape the KIT receptor at the molecular and cellular levels.^18^ The cytoplasmic mutations in the JM domain and the TKD have been proposed to disrupt JM domain-mediated autoinhibition and favor an active kinase domain conformation.^19^ Because KIT has been proven to be a dominant oncogene in Several cancers, including CML, the tyrosine kinase inhibitors (TKIs) imatinib, nilotinib, and bosutinib have been successfully used in the clinic to target oncogenic KIT-driven cancers therefore decreasing significantly,^20^ The With drug (WD) group is characterized by T350G (14% total) and multiple substitution patterns at position 404 As for that, deletion mutations appeared in only two samples (C1, C18), representing 4% of the total. Deletion mutations (even if they constitute only 4%) are often more serious than substitution mutations. Because it can lead to a frameshift mutation, which alters the entire amino acid sequence of the KIT protein downstream of the deletion point.^21^ Sample C1 recorded three deletion mutations at close positions (486, 521, 529), indicating a significant structural defect in this part of the gene in this particular individual.^22^

Despite these encouraging results, the relatively small size of the samples is considered the main limitation of this study. More research with larger sample size is still necessary before obtaining a definitive answer to the biological and clinical significance of KIT mutations in the progression of the CML disease.

## 4 Conclusion

A mutational profile of the KIT gene was investigated in both CML patients and healthy controls in this exploratory study. After the gene area was isolated and amplified by PCR, mutations were examined using Sanger sequencing and bioinformatics tools. A total of 27 mutations were detected. The variants G753C, G538C, A415T, and A404G were the most prevalent, whereas G814C, G858T, G876T, and G880A were only identified in the control cohort. Notably, several distinct mutations identified in the Iraqi community indicate particular genetic polymorphisms. Deletion mutations were also identified which were predominantly occurring in the phosphorylation site areas of the KIT. These mutations in the JM, CA, and Ph domain regions may play an important role in the development of CML. Mutations have, in fact, an independent prognostic impact on the natural course of localized, untreated Ex9 and Ex11 belonging to the same group, with the worst prognosis. With the worst prognosis. In addition, the KIT mutant in JM reign remains without a known FDA-approved optimal therapy, and future studies should focus on new treatment strategies for these patients with targetable mutations. Further understanding of the mechanisms of acquired resistance and the molecular basis of each CML subtype drug will aid in the development of the next generation of targeted therapies. Our preliminary data showed that the high BMI may heighten disease susceptibility. Despite the Philadelphia chromosome being the main cause, obesity contributes to inflammation through fat cell-derived cytokines, potentially affecting bone marrow and promoting cancer cell growth. Additionally, found mutations could interfere with the efficacy of TKIs and their necessary dosages.

## CRediT authorship contribution statement

**Asrar K. Salman:** Writing – review & editing, Writing – original draft, Visualization, Methodology, Investigation, Data curation, Conceptualization. **Ahmed E. Dhamad:** Writing – review & editing, Supervision, Project administration, Methodology, Conceptualization, Data curation, Formal analysis, Investigation, Resources, Software, Validation, Visualization.

## Funding

This research was funded by authors.

## Declaration of competing interest

The authors declare that they have no known competing financial interests or personal relationships that could have appeared to influence the work reported in this paper.
